# Plant-Based Nutrition Alleviates Gastrointestinal Symptoms and Enhances Protein Intake during GLP-1 Therapy

**DOI:** 10.1016/j.cdnut.2026.109393

**Published:** 2026-06-08

**Authors:** Nicole A Withrow, Esther E Hsueh, Thomas Wolever, Alexandra L Jenkins, Janice Campbell, Andreea Zurbau, Robert McMahon, Christina J Valentine

**Affiliations:** 1 Kate Farms, Goleta, CA, United States; 2 INQUIS Clinical Research, Toronto, Ontario, Canada; 3 Seven HIlls Strategies, Beaverton, OR, United States

**Keywords:** glucagon-like peptide-1 receptor agonist, tolerance, gastrointestinal, plant-based formula, high protein

## Abstract

**Background:**

Glucagon-like-peptide receptor agonists (GLP-1RA), such as semaglutide, are widely used for weight management but often lead to gastrointestinal (GI) side effects, which compromise nutritional intake and may increase the risk of muscle loss. This study explores whether a plant-based high-protein nutrition shake (PBHPNS) can support acceptance, tolerance, and nutrient adequacy in individuals on GLP-1RA therapy.

**Objective:**

This study aimed to determine whether a plant-based nutrition approach with increased protein and fiber would improve acceptance, tolerance, and nutrient intake of individuals on GLP-1RA therapy.

**Methods:**

This single-arm, open-label pilot study included adults (BMI >27 kg/m^2^) on GLP-1RA therapy for ≥4 wk. Participants consumed 1 to 2 servings of the PBHPNS daily for 7 d. Acceptance, likability, and GI symptoms were assessed at baseline, acute postconsumption (2 h), and postintervention. Two 24-h dietary recalls were collected.

**Results:**

Twenty participants completed the study, and there were no dropouts (13 Ozempic, 7 Wegovy). Acute GI symptoms improved significantly 2 h after the first PBHPNS consumption, particularly for abdominal bloating and constipation (*P* < 0.05). No statistically significant changes in GI symptoms were seen over the treatment period. Over the intervention period, participants significantly increased protein and fiber intake, averaging 1.7 shakes/d, and ranked them likable on a standard scale.

**Conclusions:**

The PBHPNS effectively addresses the nutritional gap in GLP-1RA users by significantly increasing protein and fiber intake without exacerbating GI symptoms. There is significantly less bloating and constipation immediately after consumption and notable trends in improved GI symptoms throughout the 7 d. This study suggests that a clinically designed, low-volume PBHPNS may mitigate the nutritional risks of muscle loss and GI distress associated with GLP-1RA–induced anorexia and gastric delay. Also, additional studies are required because of this being a pilot study.

This study is registered at clinicaltrials.gov at NCT07096739.

## Introduction

The emergence of glucagon-like peptide-1 receptor agonists (GLP-1RAs) has revolutionized the treatment landscape for type 2 diabetes and obesity. By emulating endogenous hormones secreted by enteroendocrine L-cells to enhance glucose-dependent insulin secretion and delay gastric emptying, GLP-1RA agents achieve clinically significant weight reduction ranging from 8% to 18% in real-world and clinical settings [[1], [2], [3], [4], [5]]. However, the therapeutic efficacy of GLP-1RA therapy is frequently undermined by a “Nutrition Paradox”: although high-protein and high-fiber intakes are physiologically needed to maintain metabolic health during rapid weight loss, the drug’s primary mechanism of action—gastric delay and anorexia—often renders traditional dietary adherence extremely difficult [1].

This paradox poses significant clinical challenges to muscle health. Evidence indicates that individuals on GLP-1RA therapy often reduce total energy intake by 16% to 39%, leading to a substantial loss of lean body mass (LBM) [6]. In some cohorts, 20% to 50% of total weight loss is attributable to skeletal muscle rather than adipose tissue, increasing the risk of sarcopenic obesity and metabolic rate depression [6,7]. To mitigate this, current guidelines recommend higher protein intake of 1.2 to 1.6 g/kg/d combined with resistance training [5]. Yet, patients frequently encounter gastrointestinal (GI) disturbance; the prevalence of nausea, vomiting, and bloating is high, with treatment discontinuation rates reaching 50% to 67% at 1 y and ≤85% at 2 y, primarily due to GI adverse events [8,9].

The plant-based high-protein nutrition shake (PBHPNS) evaluated in this study was purposefully engineered to resolve these physiological demands. Recognizing that high-volume solid meals aggravate the postprandial fullness associated with delayed gastric emptying, the PBHPNS offers a high-density, low-volume delivery approach. The formulation utilizes pea-protein isolate, which provides a complete amino acid profile (PDCAAS 1.0) while remaining hypoallergenic and potentially easier to digest than dairy-based proteins, which can exacerbate GI sensitivity in some obese populations [[10], [11], [12]].

Importantly, the PBHPNS is also designed to address the fiber-tolerance challenges commonly observed in GLP-1RA users. Although dietary fiber is necessary for mitigating medication-associated constipation, rapidly fermentable fibers may contribute to gas production and bloating symptoms that many patients seek to avoid. To address this, the formulation incorporates a fiber matrix composed of yellow pea, oat, agave inulin, acacia gum, and locust bean gum. This proprietary blend is designed to support staged fermentation along the GI tract. By combining fibers with varying fermentation rates across the proximal and distal colon, the formulation aims to reduce rapid gas accumulation that can trigger acute bloating while maintaining adequate fermentative and mechanical stimulation to support regular bowel function [[13], [14], [15]]. This study evaluates whether consistent intake of this medically designed, plant-based intervention can alleviate GI symptoms while addressing nutritional gaps in individuals undergoing GLP-1RA therapy. We hypothesized that regular consumption of the PBHPNS would be associated with improvements in GI symptoms and increased dietary protein intake in this population.

## Methods

This was a prospective single-arm, open-label pilot study. The study was conducted in accordance with the Declaration of Helsinki, and informed consent for participation was obtained from all subjects before their involvement in the study. The protocol and consent form were approved on 10 July 2025 by the Advarra Institutional Review Board (IRB# Pro00088191). The study was submitted to clinicaltrials.gov on 24 July 2025 and posted on 31 July 2025. Screening for eligibility lasted from 30 July to 5 September 2025, and the first and last study visits occurred on 7 August and 19 September 2025, respectively.

### Study participants

Participants were recruited from a pool who had previously participated in studies at INQUIS Clinical Research. INQUIS is a well-established clinical research organization located in Toronto, Canada. The inclusion criteria included healthy individuals, no acute illnesses or recent hospitalizations, aged 18 to 75 y with a BMI >27.0 kg/m^2^ and on an established GLP1-RA injection (e.g., Ozempic, Wegovy) for ≥4 wk before screening. Participants were receiving semaglutide injections, either Ozempic or Wegovy formulations; both are commonly used in clinical practice for glycemic control and weight management. Given the exploratory, pilot nature of the study, we aimed to include a broad representation of individuals using GLP-1RAs rather than limiting the sample to a BMI category or medical diagnosis. Additionally, participants needed an active email address, daily internet access via an electronic device, and the ability to complete a daily electronic diary.

### Study procedures

Before screening, participants had the study procedures explained to them and were given the opportunity to read the consent form and ask questions before consent was obtained. They could either sign the consent form then, refuse to participate, or take a copy of the consent form home to consider their participation.

Each participant had a total of 3 clinic visits. The screening visits occurred on the same day as the informed consent procedure or later, depending on the participant’s preference. Potential participants were screened by undergoing a medical history review, a medication and supplement assessment, and measurements of height and weight. A 3-d run-in period preceded the second clinic visit. During this period, participants completed the GI symptom questionnaire to collect baseline information. During the second visit, participants completed the GI symptom questionnaire again, reviewed their medications and medical history, and consumed a PBHPNS within a 20-min period. The GI symptom questionnaire was completed again 2 h after consuming the shake. Additionally, a 24-h dietary recall was conducted. They were instructed to consume a minimum of 1 and a maximum of 2 PBHPNS daily for 7 d. The PBHPNS was an organic pea-protein plant-based formula that contained 160 calories, 25 g of protein, 4 g of fat, 9 g of carbohydrate, and 6 g of mixed fiber per 325 mL and was available in chocolate and strawberry flavors. The mixed fiber blend was comprised fibers intrinsic to pea protein, agave inulin, acacia fiber, and oat hull fiber, and included 4 g of soluble fiber and 2 g of insoluble fiber. The pea protein was derived from yellow peas, and the shake provided complete protein with the addition of amino acids.

At home, participants consumed 1 to 2 PBHPNS daily and completed the GI symptom questionnaire on days 4, 6, and 7. On day 8, participants returned to the clinic, where their medical history, medication, height, and weight were measured, and supplement use was reviewed, along with a final GI symptom questionnaire being completed.

### Statistical analysis

Statistical analyses of symptom scores were performed by paired *t*-tests and chi-square tests; the criterion for significance was 2-tailed *P* < 0.05. Because this was a pilot study, we did not correct for multiple comparisons.

Paired *t*-tests: for the acute consumption study, the means of the paired scores before and 2 h after consuming the investigational product on day 1 were compared; for the chronic consumption study, the means of the scores on the 3 run-in days were compared with the mean of the scores on days 4, 6, and 8.

For the chi-square tests, the scores 2 h after consuming the product, and the mean scores for the 3 treatment days for each symptom, respectively, were ranked as being lower, the same, or higher than the score before consuming the product, or the mean score during the run-in period. The number of lower scores during treatment (L) and the number of higher scores during the treatment period (H, the number of the same scores was ignored) were compared with (L + H) / 2 by chi-square test (e.g., 11 and 5 compared with 8 and 8). Because the number of cases within some of the cells being compared was often low, we also used Fisher’s exact test and report the results of both tests.

Energy intake (kcal) and the intakes of fat, protein, and carbohydrate (expressed as grams and % energy) and dietary fiber (expressed as grams and g/1000 kcal) were compared at baseline and after the 7-d treatment period by paired *t*-test. The correlations between intakes and symptom scores on day 7 were also assessed. The criterion for significance was *P* < 0.05.

The number of participants within each of the 9 likeability scores for each question was tabulated. However, with only 20 participants, most of the 9 cells contained <4 responses; because the chi-square test is unreliable unless there are ≥4 responses in each cell, the results were collapsed into 3 categories for statistical analysis to determine if more people liked rather than disliked the PBHPNS. The correlation of these scores with each other and with the symptoms scores was assessed, and the criterion for significance was *P* < 0.05.

## Results

### Participants

Of 24 individuals screened, 3 did not meet recruitment criteria; 2 had not been on GLP-1 medications for a minimum of 30 d and 1 met the criteria but was not enrolled because of scheduling conflicts, and there were no dropouts. The 20 participants who completed the study comprised 16 females and 4 males of varied race (*n* = 11 Caucasian, *n* = 3 Asian, *n* = 3 Hispanic, *n* = 2 Black, and *n* = 11 Middle Eastern) (Table 1), aged [mean 46.8 ± 120 (24–64)] y, with BMI (mean 35.4 ± 7.8 kg/m^2^, 1 = 27.5, 2 = 29.4, 17 >30). Enrollment of GLP-1RA users was guided by prescribing trends, with women comprising >75% of the users when compared with men. This study was structured to mirror this distribution.

**TABLE 1:** Summary of participant demographic characteristics (*n* = 20).

Current age (y): min–max, mean (SD)	24–64, 46.8 (12.0)
	*n* (%)
Current age range (y)	
20–29	1 (5)
30–39	3 (15)
40–49	7 (35)
50–59	7 (35)
60–69	2 (10)
Gender	
Female	16 (80)
Male	4 (20)
Race/ethnicity	
Asian	3 (15)
Black	2 (10)
Caucasian	11 (55)
Hispanic	3 (15)
Middle Eastern	1 (5)

### Medical history and medication use

Across the 20 participants, the most reported medical diagnoses were endocrine disorders (*n* = 24, 3 overweight/17 obesity, 3 hypothyroidism, and 1 type 2 diabetes), allergies and sensitivities (*n* = 18), and GI (*n* = 12) (Table 2). All participants were on semaglutide injections (13 participants were using Ozempic and 7 were using Wegovy) (Table 3).

**TABLE 2:** Summary of diagnosed GI, endocrine, and allergy medical history (*n* = 20).

GI	*n* (%)
Heartburn	3 (15)
Constipation	3 (15)
Gallstone	2 (10)
Hemorrhoids	2 (10)
Gastric reflux	2 (10)
Endocrine	
Overweight	3 (15)
Obesity	17 (85)
Hypothyroidism	3 (15)
Type 2 diabetes	1 (5)
Allergies or sensitivities	18 (90)

Abbreviation: GI, gastrointestinal.

**TABLE 3:** Participant-reported medication and supplement use by indication (*n* = 20).

	*n* (%)
Weight loss	
Ozempic	13 (65)
Wegovy	7 (35)
Hypertension	2 (10)
Hypercholesterolemia	1 (5)
GI	
Nausea	1 (5)
Heartburn	3 (15)
Constipation	3 (15)
Reflux	2 (10)
General health	
Multivitamin	9 (45)
Omega 3	5 (25)
Vitamin B12	3 (15)
Vitamin C	1 (5)
Vitamin D	5 (25)
Magnesium	1 (5)
Calcium	3 (15)
Mushroom complex	1 (5)
Probiotic	2 (10)
Turmeric	2 (10)
Collagen	1 (5)
Melatonin	1 (5)
Apple cider vinegar	1 (5)
Cranberry complex	1 (5)

Abbreviation: GI, gastrointestinal.

### Acute GI symptom score (2-h challenge)

Acute GI symptoms scores were collected on day 1 of the intervention period when participants were given the first serving of the PBHPNS. There was significantly less abdominal bloating and constipation, and a significantly lower composite score of all symptoms and acute symptoms 2 h after PBHPNS compared with fasting levels (Table 4). There was a trend toward a reduction in nausea, abdominal discomfort, gas/flatulence, and diarrhea. There were no episodes of vomiting during the 2-h test period.

**TABLE 4:** Acute symptoms (bloating, discomfort, gas/flatulence, nausea, vomiting): mean scores for before and 2 h after HPNS intake.

GI symptom score	Severity scores^1^			Change in severity score^2^			
	0 min	120 min	*P*^3^	Lower	Same	Higher	*P*^4^
Abdominal bloating	0.50 ± 0.11	0.25 ± 0.10	0.021	5 (25%)	15 (75%)	0 (0%)	0.068 (0.083)
Abdominal discomfort	0.45 ± 0.14	0.25 ± 0.12	0.10	5 (25%)	14 (70%)	1 (5%)	0.22 (0.24)
Gas/flatulence	0.40 ± 0.13	0.20 ± 0.12	0.10	5 (25%)	14 (70%)	1 (5%)	0.22 (0.24)
Nausea	0.35 ± 0.13	0.20 ± 0.09	0.083	3 (15%)	17 (85%)	0 (0%)	0.16 (0.20)
Vomiting	0.00 ± 0.00	0.00 ± 0.00	—	0 (0%)	20 (100%)	0 (0%)	—
Diarrhea	0.05 ± 0.05	0.00 ± 0.00	0.33	1 (5%)	19 (95%)	0 (0%)	0.41 (0.50)
Constipation	0.25 ± 0.10	0.00 ± 0.00	0.021	5 (25%)	15 (75%)	0 (0%)	0.068 (0.083)
Acute symptoms composite score (excluding diarrhea and constipation)	1.70 ± 0.39	0.90 ± 0.30	0.004	8 (40%)	12 (60%)	0 (0%)	0.021 (0.038)

Symptoms were scored as follows: 0 = none, 1 = mild, 2 = moderate, 3 = severe.

Abbreviations: GI, gastrointestinal; HPNS, high-protein nutrition shake.

1 Values are means ± SEM of the mean of the severity scores at 0 min and 120 min in 20 subjects.

2 Values are number (%) of subjects with severity score at 120 min less than at 0min (Lower), the same as at 0 min (Same) or greater than at 0 min (Higher).

3 p-value from a paired t-test comparing scores at 0 and 120 min.

4 p-values for a chi-squared test (Fisher's exact test) comparing of the observed number of subjects with a lower score vs a higher score with and equal number lower vs higher (e.g., for Composite Score, 10, 0 was compared to 5, 5; in this analysis subjects with the same score are ignored)

### Chronic GI symptoms score

There were no significant differences between the severity of individuals or composite scores before (mean of 3 baseline days) compared with during the intervention period (mean of 3 d during the 1-wk treatment) (Table 5). However, there were notable trends; during treatment, 2 to 3 times more people reported less, as compared with more, abdominal bloating (*n* = 8 compared with *n* = 4), abdominal discomfort (*n* = 8 compared with *n* = 3), and constipation (*n* = 9 compared with *n* = 3) (Table 5). Similarly, during treatment, 1.5 to 2 times more people had a lower, as compared with a higher, composite score for all symptoms (*n* = 11 compared with *n* = 7) (Table 5).

**TABLE 5:** Chronic symptoms (all symptoms): mean scores over 3 d before and on days 4, 6, and 8 during the 1-wk treatment period.

GI symptom score	Severity scores^1^			Change in severity score^2^			
	Before	During	*P* value^3^	Lower	Same	Higher	*P* value^4^
Abdominal bloating	0.63 ± 0.12	0.42 ± 0.11	0.085	8 (40%)	8 (40%)	4 (20%)	0.41 (0.23)
Abdominal discomfort	0.62 ± 0.16	0.38 ± 0.10	0.090	8 (40%)	9 (45%)	3 (15%)	0.27 (0.10)
Gas/flatulence	0.53 ± 0.11	0.63 ± 0.14	0.52	6 (30%)	8 (40%)	6 (30%)	1.00 (0.32)
Nausea	0.23 ± 0.10	0.22 ± 0.08	0.83	3 (15%)	15 (75%)	2 (10%)	0.75 (0.24)
Vomiting	0.00 ± 0.00	0.02 ± 0.02	0.33	0 (0%)	19 (95%)	1 (5%)	0.41 (0.50)
Diarrhea	0.07 ± 0.05	0.12 ± 0.04	0.33	2 (10%)	14 (70%)	4 (20%)	0.56 (0.38)
Constipation	0.47 ± 0.14	0.20 ± 0.07	0.072	9 (45%)	8 (40%)	3 (15%)	0.21 (0.16)
Chronic symptoms composite score (all symptoms)	2.55 ± 0.47	1.98 ± 0.38	0.21	11 (55%)	2 (10%)	7 (35%)	0.50 (0.21)

Abbreviation: GI, gastrointestinal.

1 Values are means ± SEM of the mean severity scores over 3 d before and on days 4, 6, and 8 during the 1-wk treatment period in 20 subjects.

2 Values are number (%) of subjects with a mean severity score of 3 days during less than a mean severity score of 3 days before (Lower), the same as a mean severity score of 3 days before (Same) or greater than a mean severity score of 3 days before (Higher).

3 p-value from a paired t-test comparing mean scores before and during.

4 p-values for a chi-squared test (Fisher's exact test) comparing of the observed number of subjects with a lower score vs a higher score with and equal number lower vs higher (e.g., for Composite Score, 10, 0 was compared to 5, 5; in this analysis subjects with the same score are ignored).

### Nutrient intake

There was a significant increase in total protein and total dietary fiber intake (Table 6). There was a significant reduction in energy-adjusted carbohydrate intake. The reduction in carbohydrate intake (9% of energy) was likely due to an increase in protein, and the total protein (44 ± 8 g) and total dietary fiber (9.7 + 2 g) correspond with ∼1.7 HPNS/d. There was no change in the percent of calories from fat intake between day 7 and baseline.

**TABLE 6:** Energy, macronutrient, and fiber intake at baseline and day 8 of HPNS consumption.

24-h recall data	Baseline (SV 2)	Day 8 (SV 3)	*P* value (absolute intake/energy adjusted)
Calories	1419 ± 122	1530 ± 135	ns
Carbohydrates (g)	156 ± 15	135 ± 17	ns/0.005
Fat (g)	60 ± 122	65 ± 135	ns/ns
Protein (g)	66 ± 4	110 ± 8	<0.001/<0.0001
Fiber (g)	12 ± 1	22 ± 1	<0.001/<0.001

Data presented as mean ± SEM. Differences between baseline and day 8 were assessed using 2-tailed paired *t*-test.

Abbreviations: GI, gastrointestinal; HPNS, high-protein nutrition shake; ns, not significant; SV, study visit.

### Likeability

Among 20 participants, 12 consumed both flavors, 6 consumed only chocolate, and 2 consumed only strawberry. The overall product likeability score was 6.2 ± 0.5 on a 9-point scale, indicating “like slightly” to “like moderately.” There was no significant difference in likeability between flavors and no significant differences in nutrient intake changes from baseline to day 7 between those who liked compared with disliked the products. Participants consumed on mean 1.7 PBHPNS/d, with a minimum of 1 required each day. Likeability was positively correlated with protein intake at day 7 (*r* = 0.595, *P* = 0.006). No significant correlations were found between likeability and GI symptom scores.

## Discussion

To our knowledge, this study is the first to investigate GI tolerance and likeability of a plant-based high-protein nutritional shake specifically among individuals undergoing therapy with GLP-1RAs. Our findings demonstrate that the intervention successfully addressed a nutritional vulnerability common in this population, significantly increasing total protein and fiber intake without exacerbating, even mitigating, GI symptoms.

### Bridging the gap: nutrient density and LBM preservation

The achievement of ∼110 g of daily protein is clinically meaningful, as it is generally consistent with the 1.2 to 1.6 g/kg/d threshold required to preserve LBM during rapid weight loss [5]. For a GLP-1RA user experiencing drug-induced appetite suppression and slowed gastric emptying, achieving this protein intake through whole foods can be physically challenging because of early satiety onset. This nutrient density—the ability to deliver high-density nutrients in a low-volume format—allows for the altered motility of the GLP-1RA–treated gut. By ensuring adequate protein delivery, the PBHPNS may serve as a critical tool in preventing sarcopenic obesity, ensuring that weight loss is primarily derived from adipose tissue while metabolic rate and physical function are maintained [[16], [17], [18], [19]].

### Possible mechanism: a prebiotic effect coupled with staged fermentation

Perhaps the most notable finding was the significant acute improvement in abdominal bloating and constipation within 2 h of PBHPNS consumption. Typically, fiber-enriched products might be expected to increase temporary bloating. However, the PBHPNS is designed with a “staged fermentation” strategy. The blend of agave inulin, acacia gum, pea fiber, and oat hull fiber targets different regions of the colon. Acacia gum, for instance, ferments slowly in the distal colon, reducing the risk of rapid gas buildup in the proximal gut where GLP-1RA–induced delay is most pronounced [13].

The significant reduction in bloating may be explained in part by a bacterial pathway known as the bifidus shunt. Prebiotic fibers like inulin and acacia gum selectively stimulate the growth of *Bifidobacteria*. Unlike many other colonic bacteria that produce hydrogen or methane as byproducts of carbohydrate fermentation, *Bifidobacteria* utilize the fructose-6-phosphate shunt, which yields metabolites such as acetate (a short-chain fatty acid) and lactate, which can serve as an important intermediate that can be further metabolized by other gut microbes to generate butyrate [20,21]. This metabolic shift might alleviate the pressure that causes bloating but also provides short-chain fatty acids that enhance gut barrier function and reduce local inflammation, which is often elevated in individuals with obesity [22,23].

### Alleviating GI disturbance to support adherence

The clinical importance of these findings may extend to drug adherence. GI adverse events are documented as the leading cause of GLP-1RA discontinuation, with real-world persistence rates dropping precipitously over the first 2 y [8,9]. By providing a nutrition source that not only is well tolerated but also actively improves acute symptoms like constipation and bloating, clinicians can potentially reduce “symptom-driven discontinuation.” Alleviating eating-related discomfort is essential for sustaining long-term metabolic outcomes in patients who must remain on these therapies indefinitely [8].

### Limitations and future directions

The limitations of this study include a relatively small sample size and short duration, which may limit the generalizability of the results and the ability to detect long-term effects in chronic GI symptoms. Furthermore, several confounding factors could have influenced the observed outcomes. Participants were on varying dosages of GLP-1RA medication, which is known to contribute to the severity and prevalence of GI symptoms and could have potentially impacted the intake of the PBHPNS. The medical history of participants also revealed pre-existing GI diagnosis, including reflux and constipation, which were included in the top 5 most common diagnoses. Participants also reported the onset of these diagnoses for >1 y before the study’s initiation, except for 1 mild case of constipation starting earlier in the study. Variations in participants’ pre-existing conditions (e.g., postmenopausal women and BMI categorization) and GI symptom histories introduce heterogeneity that may influence their responses to both GLP-1RA treatment and the nutritional shake. Moreover, differences in the rate and magnitude of individuals’ weight loss represent an additional confounding factor that can affect GI symptoms patterns and overall nutritional intake [24].

Future research should aim to address these limitations by employing larger sample sizes and incorporating randomized controlled trial designs to minimize potential placebo effects and to more rigorously evaluate the effects of this product compared with other oral nutrition supplements. Additionally, future studies should consider stratification by BMI category and underlying medical diagnoses. Longer follow-up periods to evaluate the sustained impact of the PBHPNS on GI tolerance, nutrition status, lean body preservation, and overall patient outcomes in GLP-1RA users would be beneficial. Studies that control for GLP-1RA dosage, indications for use, account for pre-existing GI conditions, and monitor the rate and magnitude of weight loss would assist in a better understanding of the clinical benefits and applicability of the PBHPNS.

In conclusion, the PBHPNS was well accepted and increased protein intake in GLP-1RA users. The formulation supports the physiological needs of the patient while mitigating the GI barriers that threaten therapeutic success. These results may provide a first foundation for integrating targeted plant-based nutrition into the standard of care for obesity management.

## Author contributions

The authors’ responsibilities were as follows – NAW, TW, AJ, JC: conceived and designed the research; JC: conducted the research; NAW, AZ, JC: provided essential materials necessary for the research; AZ, TW: performed statistical analysis; NAW, EH: wrote the paper, assisted in writing and editing the manuscript; RM: assisted in writing and editing the paper, assisted in writing and editing the manuscript; CJV: conceived and designed the research, had primary responsiblity for editing final content, had primary responsibility for final content; and all authors: read and approved the final manuscript and agree to be fully accountable for ensuring the integrity and accuracy of the work. CJV:conceived and designed the research, had primary responsiblity for editing final content. NAW, EH, RM assisted in writing and editing the manuscript.

## Data availability

Data described in the manuscript, code book, and analytic code will be made available upon request pending (e.g., application and approval, payment, other).

## Declaration of Generative AI and AI-assisted Technologies in the Writing Process

During the preparation of this work, the authors used Gemini to assist in editing. After using this tool/service, the authors reviewed and edited the content as needed and take full responsibility for the content of the published article.

## Funding

Funding to conduct the study was provided by Kate Farms, Inc.

## Conflict of interest

NW, EH, and CJV are employed by Kate Farms, Inc., Goleta, CA. RM is a consultant for Kate Farms, Inc., Goleta, CA.
